# Sarcoptic Mange in Reintroduced Red Foxes (*Vulpes vulpes*) in South Korea: Case Histories, Clinical Assessments, Treatments, and Pathological Findings

**DOI:** 10.3390/ani15101491

**Published:** 2025-05-21

**Authors:** Sook-Jin Lee, An-Na Lee, Eun-Bin Shin, Min-Sung Kim, Hyoung-Jin Kim, Doo-Hyun Han, Yong-Sik Jo, Jin-Suk Ahn, Seung-Hoon Chea, Chang-Min Jeong, Hee-Yeon Lee, Seong-Geun Bae, Jeong-Jin Yang

**Affiliations:** 1 National Park Institute for Wildlife Conservation, Korea National Park Service, 33, Sobaekro 2481beon-gil, Yeongju 36015, Republic of Korea; mfungus5@knps.or.kr (S.-J.L.); lananna@knps.or.kr (A.-N.L.); 23bean@knps.or.kr (E.-B.S.); kms0322@knps.or.kr (M.-S.K.); tekken3ttt@knps.or.kr (H.-J.K.); gks8760@knps.or.kr (D.-H.H.); cho0122@knps.or.kr (Y.-S.J.); ajs267@knps.or.kr (J.-S.A.); cshswww@knps.or.kr (S.-H.C.); 2020059128@knps.or.kr (C.-M.J.); lhy0131@knps.or.kr (H.-Y.L.); last1127@knps.or.kr (S.-G.B.); 2 National Park Institute for Wildlife Conservation, Korea National Park Service, Hwaeomsaro 402-31, Gurye 57616, Republic of Korea

**Keywords:** blood test, red fox, *Sarcoptes scabiei*, sarcoptic mange, South Korea, *Vulpes*

## Abstract

Sarcoptic mange is a highly contagious and often lethal parasitic skin disease caused by the mite *Sarcoptes scabiei*, which is frequently reported in red foxes (*Vulpes vulpes*). However, there is a lack of documented cases and treatment strategies for sarcoptic mange in reintroduced red fox populations, particularly in South Korea. This study describes 27 cases of sarcoptic mange outbreaks in 26 red foxes reintroduced into South Korea between 2019 and 2024. Of these, 15 foxes were rescued alive, while 12 were found dead. Blood tests of the surviving animals (*n* = 15) showed significant leukocytosis, anemia, decreased albumin levels, increased globulin levels, elevated blood urea nitrogen levels, and decreased creatinine levels, indicative of a chronic wasting infection. Treatment with ivermectin or fluralaner resulted in complete recovery in 12 of 15 animals, while three animals died during treatment. Necropsy of the deceased animals (*n* = 12) revealed characteristic skin lesions, such as alopecia, hyperkeratosis, epidermal hyperplasia, and dermatitis. The study findings highlight the importance of long-term monitoring and active, continuous treatment of sarcoptic mange, a major threat, for the stable re-establishment of reintroduced foxes in South Korea.

## Simple Summary

The red fox (*Vulpes vulpes*) became extinct in South Korea, and a restoration project through reintroduction of these foxes has been ongoing since 2012. Sarcoptic mange, a parasitic skin disease, has been a major threat to reintroduced foxes since the project’s initiation. Between 2019 and 2024, 27 cases of sarcoptic mange infections were identified in 26 red foxes. In 15 of these cases, where the foxes were rescued alive, clinical assessments, including physical examinations, blood tests, and treatments, were conducted, and their results were documented. For 12 foxes found dead, pathological findings were confirmed through necropsy. This study presents the first report of clinical cases of sarcoptic mange infection in reintroduced red foxes in South Korea and aims to contribute to improving individual survival rates and promoting the establishment of healthy red fox populations.

## 1. Introduction

Sarcoptic mange is a highly contagious skin disease in mammals caused by the mite *Sarcoptes scabiei*. Infections have been reported in 148 mammalian species, including wild animals [1]. Among these, the red fox (*Vulpes vulpes*) is highly susceptible to sarcoptic mange across a wide geographic range [2,3,4,5,6,7]. Factors such as host population size, social behavior, movement patterns, and habitat influence the transmission of sarcoptic mange. The red fox is reported to be a species in which both direct transmission related to high population density and indirect transmission associated with shared dens are possible [5,8].

Sarcoptic mites create tunnels within skin layers, thereby disrupting the protective barrier of the skin and causing different clinical symptoms depending on the host’s immune status [9,10]. The initial clinical signs, such as pruritus, erythema, papule formation, and seborrhea, manifest as a hypersensitivity reaction to the mites. As the infection progresses, the skin becomes crusted, and alopecia, lichenification, and hyperpigmentation develop [9,10]. Eventually, the general condition deteriorates, leading to severe dehydration, debilitation, and, ultimately, death [11,12]. Histologically, experimentally infected red foxes exhibited parakeratotic and orthokeratotic hyperkeratosis, acanthosis, and dermal infiltration by eosinophils and mast cells, especially in the vicinity of mites; when epidermal erosion and crusting are present, neutrophils and plasma cells may become more prominent. Hyperkeratosis and epidermal hyperplasia intensified as the infection progressed [13].

Sarcoptic mange has varying effects on red fox populations. In cases of sporadic outbreaks in stable populations, as observed in North America, the overall population size is not significantly affected [7]. However, even in stable populations, epidemics can lead to a substantial decline. For example, in the UK, a sarcoptic mange epidemic resulted in a rapid decline of over 95% in the fox population within 2 years [3,14]. Similarly, in Norway and Sweden, the first outbreak led to a 50–90% decrease in fox numbers [15,16,17]. In fragmented or naïve populations, sarcoptic mange can be lethal enough to cause local extinctions, as evidenced by the extinction of the red fox population on Bornholm Island in Denmark following a sarcoptic mange outbreak, potentially necessitating individual treatment [10,18].

The most commonly used medication for treating sarcoptic mange in wild animals is ivermectin injection [19]. Recently, there have been reports of effective treatments using fluralaner in individual wild animals [20,21,22]. However, in red foxes, only one report has described treatment with ivermectin in naturally infected individuals [23]. Complete blood count (CBC) and serum chemistry values of mange-infected wildlife canids have been compared to those of healthy individuals to assess physiological parameter alterations and aid in medical management [24,25,26]. However, no blood test results have been reported for naturally infected red foxes, only those from experimentally infected individuals [13].

In South Korea, the population of red foxes sharply declined and was reported to have become extinct after 1960 due to habitat destruction and overhunting for fur [27]. Consequently, in 1997, the Ministry of Environment designated the red fox as a critically endangered species. Following the discovery of the last wild fox carcass in Yanggu, located in the northern region of South Korea, in 2004, the need for a restoration project was highlighted. The red fox restoration project in South Korea was launched with the primary goal of preventing the extinction of red foxes and establishing a population of more than 50 individuals. The project began in 2012 with the introduction and release of red foxes genetically close to the native Korean fox from the northeastern region of China into the Sobaeksan National Park area located in the central region of South Korea [28,29].

In South Korea, sarcoptic mange has been reported as an endemic disease affecting not only humans but also domestic animals such as dogs and pigs, as well as wildlife species like raccoon dogs and long-tailed gorals, according to previous studies [30,31,32]. However, to date, there have been no reported clinical cases of sarcoptic mange infections in wild red foxes in South Korea, nor any reports of outbreaks in reintroduced wildlife. Recently, the increase in sarcoptic mange infections among reintroduced wild red foxes has emerged as a significant threat to their survival. This study presents the first report of sarcoptic mange infections in reintroduced red foxes in South Korea, based on clinical assessments, treatments, and pathological findings obtained through postmortem examinations. By establishing diagnostic and treatment strategies for infected individuals, this study aims to contribute to improving individual survival rates and promoting the formation of healthy red fox populations.

## 2. Materials and Methods

### 2.1. Pre-Release Management

All introduced and captive-born foxes were managed in the outer enclosure of the Central Conservation Center of the National Park Institute for Wildlife Conservation, located near Sobaeksan National Park. The foxes received preventive treatment, including vaccination for distemper, hepatitis, parvovirus, parainfluenza, leptospirosis, rabies, and deworming, as part of their annual health examination. Based on the animals’ conditions (such as age and physical health), they were classified into categories for breeding, permanent captivity, or release.

Before release, a complete health examination was conducted for quarantine and preventive medication for parasites (both ecto- and endoparasites) was administered. For ectoparasite prevention, ivermectin injection (Ivomec^®^, Boehringer Ingelheim Animal Health do Brasil Ltd.a., Paulinia, Brazil) was administered; since 2019, oral fluralaner (Bravecto^®^, Intervet GesmbH, Siemensstrabe, Austria) has been used as an alternative. All foxes were fitted with GPS collars (WT300, KoEco, Daejeon, South Korea) and radio transmitters (M3620, ATS, Isanti, MN, USA) prior to release.

### 2.2. Post-Release Management

From 2012 to 2024, approximately 230 foxes were released. Their daily locations were monitored via web-based tracking and radio telemetry until the battery was depleted (1–2 years). Camera traps were installed at activity sites based on tracking data to monitor the released foxes. If clinical signs of sarcoptic mange were suspected from the camera footage or if no movement was detected for 2–3 days via tracking, direct visual observations were conducted to assess the health condition of the foxes. If the foxes were found to be in poor condition, they were captured using traps or nets. Foxes, other than those monitored after release, were found based on sighting reports from local residents. During the restoration project, all rescued foxes and carcasses were transported to the Central Conservation Center of the National Park Institute for Wildlife Conservation, Korea National Park Service.

### 2.3. Cases and Clinical Assessment

Health checks and treatments were performed on living animals, while all discovered carcasses were subjected to necropsy to determine the cause of death. The study protocol was approved by the Animal Care and Research Committee of the National Park Institute for Wildlife Conservation (NPIWC number 24-1398).

All rescued foxes were anesthetized using an intramuscular injection of a combination of medetomidine (0.05 mg/kg, Tomidin^®^, 1.0 mg/mL, Provet Veterinary Products, Istanbul, Turkey) and ketamine (2 mg/kg, Yuhan Ketamine^®^, 50 mg/mL, Yuhan, Seoul, South Korea) administered in squeeze cages. Comprehensive health assessments, including physical examinations and blood tests, were performed. Body temperature was measured using a digital thermometer inserted into the rectum. The ages of all released foxes were known in advance. For wild-born foxes, age was estimated based on body size, dental wear, and the status of growth plates observed on skeletal radiographs. Animals aged less than 1 year were classified as juveniles, while those aged over 1 year were classified as adults. During anesthesia, fluid therapy with 0.9% normal saline was administered intravenously, with the dosage adjusted according to the individual’s hydration condition. Blood samples (approximately 5 mL) were collected from the cephalic vein and divided into EDTA and lithium heparin tubes for immediate analysis. A total of 12 CBC (BC-2800Vet, Shenzhen Mindray Animal Medical Technology Co., Ltd., Shenzhen, China) and 13 serum chemistry parameters (Catalyst One, IDEXX Laboratories, Westbrook, ME, USA) were immediately analyzed from the collected samples.

**Figure 1:**
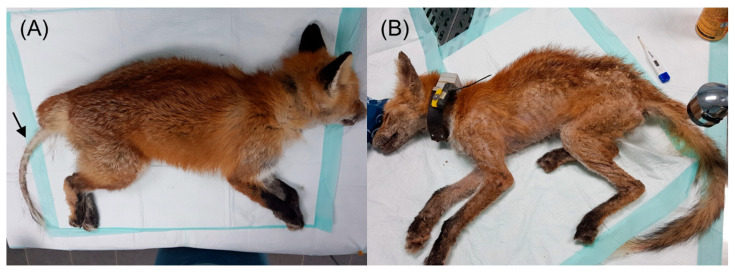
Skin lesions in red foxes (*Vulpes vulpes*) with sarcoptic mange at the National Park Institute for Wildlife Conservation: (**A**) local lesion with tail alopecia and crusting (arrow); (**B**) diffuse lesions covering more than 50% of the entire body, with severe emaciation, crusting, and alopecia throughout the body.

Sarcoptic mange infections were diagnosed based on unique clinical symptoms and the microscopic identification of mites in skin scrapings [33]. The most severe skin lesions were scraped with a scalpel from a minimum of five sites, macerated, and diluted in saline. The samples were then covered with a coverslip and initially examined under an optical microscope at 10× magnification to detect adult mites and eggs, with final confirmation at 100× magnification. If skin lesions, such as hair loss, crusting, and scaling, affected less than 50% of the body, the infection was classified as localized. If the lesions covered more than 50% of the body, it was considered diffuse (Figure 1).

All deceased foxes underwent necropsy for the visual inspection of lesions. Organs with abnormalities noted during the gross examination were fixed in 10% formalin and sent to a veterinary diagnostic laboratory (Neodin Biovet Laboratory, Guri, South Korea) for histopathological evaluation.

### 2.4. Treatment for Rescued Foxes

For the treatment of sarcoptic mange, on the day of rescue, foxes were administered ivermectin at a dose of 300 μg/kg via subcutaneous injection every week (*n* = 14) or a single oral dose of fluralaner at 250 mg (*n* = 1). Additionally, intravenous antibiotics (cefazolin sodium, 30 mg/kg) were administered to treat secondary bacterial infections. Foxes with anemia, as indicated by blood tests, were treated with iron dextran (Prolongal^®^, 10 mg/kg, IM, Elanco Animal Health Korea Co., Ltd., Ansan, South Korea). While hospitalized, they were administered the broad-spectrum oral antibiotic cephalexin (Cefaseptin^®^, Vetoquinol Korea, Koyang, South Korea) at a dose of 30 mg/kg once daily for at least 1 week. Subsequently, the foxes were re-evaluated under anesthesia at weekly intervals to monitor changes in skin lesions and overall health. Additional iron supplements and antibiotics were administered as required. Treatment was continued until the clinical signs resolved and blood values returned to normal.

### 2.5. Statistics

Blood test results of mange-infected animals on the day of rescue were statistically compared with those of healthy animals using IBM SPSS Statistics 18 software (Foster City, CA, USA). The control group consisted of healthy adult red foxes in captivity. Blood test results were obtained from regular health checkups conducted between 2019 and 2023. Control foxes were anesthetized and tested using the same equipment as that used for the infected animals. Owing to the small sample size of mange-infected individuals, normality was first tested using the Kolmogorov–Smirnov test. The results from healthy adults were assumed to be normally distributed, as the sample size exceeded 100. Data that met normality assumptions were compared with healthy adult results using Student’s t-test, while data that did not meet normality assumptions were compared using the Mann–Whitney U test. Statistical significance was considered for *p*-values less than 0.05.

Additionally, a Mann–Whitney U test was conducted to assess potential differences in the blood test results of mange-infected individuals based on age (juvenile vs. adult), sex, and the severity of skin lesions (localized vs. diffuse).

## 3. Results

### 3.1. Clinical and Pathological Findings

Between 2019 and 2024, sarcoptic mange infections were confirmed in 26 wild foxes, with a total of 27 recorded infection cases (Table 1). One fox was re-infected 302 days after release, following the resolution of its first infection (Table 1, Case 1). Of the 27 cases, 15 were identified in 14 live foxes, while the remaining 12 were found in deceased foxes. Infections were observed in juveniles and adults up to 6 years old, with no significant age-related differences. The incidence was higher in females than in males (17 vs. 10), and most infections were observed during the summer and fall (81.5% of total cases). Of the 27 cases, 21 were from monitored foxes after release, while the remaining six were from wild foxes that had not been released. The average time to confirm a mange infection after release was 345.4 days (range: 168–1211 days). The average weight of mange-infected foxes at the time of presentation was 5.03 kg (range: 3.48–6.50 kg), representing a 0.64 kg decrease from their pre-release average weight of 5.67 kg (range: 4.70–8.32 kg).

Among the 15 live-rescued foxes, seven exhibited localized skin lesions, while the remaining eight had diffuse skin lesions (Table 1, Figure 1). Localized skin lesions were most commonly found on the hind legs (especially in the hock area), followed by the tail, ear tips, muzzle, and elbows. The average body temperature of 12 animals was 38.9 °C, which is within the normal range, while three animals exhibited severe hypothermia (below 35 °C). Among the 12 carcasses with mange, two died due to trauma (car collisions and bite wounds) rather than direct mange infection, although they had localized lesions (Table 2). Except for four foxes with severe autolysis, which made accurate assessment difficult, all deceased foxes exhibited severe diffuse skin lesions. Histopathological examination revealed varying degrees of hyperkeratosis and dermatitis.

### 3.2. Blood Test Analysis

The blood test results of mange-infected wild foxes and healthy foxes in captivity were compared (Table 3). In the CBC results, the numbers of white blood cells, lymphocytes, monocytes, and granulocytes in infected foxes were significantly higher than those in healthy foxes (*p* < 0.001, *p* = 0.004, *p* = 0.002, and *p* < 0.001, respectively). Among the white blood cell components, only the proportion of eosinophils was significantly higher in infected foxes (15.5%) than in healthy foxes (8.9%) (*p* < 0.001). In terms of red blood cell parameters, the mean values for total red blood cell count, hemoglobin, and hematocrit were all significantly lower in infected foxes than in healthy foxes (*p* < 0.001), with these values being approximately two-thirds of those in healthy foxes. The average platelet count was significantly higher in infected foxes than in healthy foxes (*p* < 0.001).

The results of the serum chemistry tests showed that the total protein levels were not significantly different between infected and healthy foxes. However, the albumin levels were significantly lower in infected foxes than in healthy foxes (*p* < 0.001), whereas the globulin levels were significantly higher (*p* < 0.001). Blood glucose levels were lower in the infected group; however, the difference between the two groups was not significant (*p* = 0.739). Levels of liver enzymes, specifically alanine aminotransferase and alkaline phosphatase, were elevated in infected foxes compared to those in healthy foxes; however, the differences were not statistically significant (*p* = 0.119 and *p* = 0.246, respectively). The blood urea nitrogen (BUN) level was significantly higher in infected foxes than in healthy foxes (*p* = 0.014). Conversely, creatinine levels were significantly lower in infected foxes (*p* < 0.001). Electrolytes, such as calcium, phosphorus, sodium, potassium, and chloride, showed no significant differences between the two groups.

The blood test results of mange-infected individuals revealed no significant differences between age and sex groups. However, in foxes with diffuse skin lesions, the number of circulating lymphocytes and monocytes was significantly higher compared to those with localized lesions (*p* = 0.028 and *p* = 0.039, respectively), while the number of red blood cells, hemoglobin levels, hematocrit, and creatinine levels were significantly lower (*p* = 0.021, *p* < 0.001, *p* < 0.001, and *p* = 0.001, respectively).

### 3.3. Treatment for Rescued Foxes

Of the 15 live-rescued foxes, 12 were successfully treated, including all seven foxes with localized lesions and five of the eight foxes with diffuse lesions (Table 1). The average treatment duration was 21 days, ranging from 15 to 23 days for localized lesions and 25 to 36 days for diffuse lesions. Of the 15 rescued animals, 14 were treated with ivermectin, resulting in 11 achieving full recovery and 3 succumbing. Fluralaner was administered to one animal with a localized infection, which resulted in complete recovery. Of the 12 treated foxes, eight were re-released immediately after complete recovery, and their locations were monitored using telemetry and camera trapping, as described above. One fox with a forelimb amputation was permanently kept in captivity, and three were managed in the facility for breeding purposes.

The three foxes that died during treatment succumbed the day after rescue. One of these foxes, at the time of presentation, had both a mange infection and a pelvic fracture resulting from a car collision, while the other two had no additional health problems beyond the mange infection.

## 4. Discussion

Since the initiation of the fox release in 2012, sarcoptic mange has been the most frequent cause of morbidity among rescued wild foxes, accounting for 34.9% of all cases, excluding trauma. These results are consistent with those of previous studies. In the southeastern United States, sarcoptic mange was the leading cause of red fox rescues over a 29-year period, representing 65% of cases [2]. Similarly, at the Virginia Wildlife Center, mange was the most frequently diagnosed single disease in red foxes over a 9-year period [34]. In our study, the overall case fatality rate for mange was 55.6% (15 of 27 cases), while the mortality rate during treatment was 20% (3 of 15 cases). A study of endangered kit foxes in California over 2 years found a higher case fatality rate of 70% (12 of 15 cases) due to sarcoptic mange [35]. Red foxes do not develop immunity against reinfection with sarcoptic mange [13], and natural recovery without treatment in the wild has rarely been observed [36]. Most experimental infections result in death [11,12]. Thus, active intervention and treatment are crucial to enhance survival rates.

Our study found a higher incidence of sarcoptic mange in females, particularly during the summer and fall seasons, with no significant differences across age groups. Typically, male mammals are more susceptible to parasitic infections than females due to immune suppression by sex hormones [37]. Additionally, younger individuals are more susceptible to sarcoptic mange infections because of their less-developed immune systems and higher rates of inter-individual contact [38]. Consequently, they have high mortality rates and short survival times [3,23]. In our study, as most results were from released foxes, the disease incidence may differ from that observed in foxes living solely in the wild. Owing to the small sample size of this study, it is necessary to analyze more cases in the future to identify disease outbreak patterns in reintroduced foxes.

Animals infected with sarcoptic mange experience increased heat loss and energy demands due to hair loss, leading to nutritional imbalances [39]. A previous study reported a 15–33% decrease in body mass in red fox carcasses infected with sarcoptic mange compared to non-infected animals [23]. Similarly, in our study, red foxes infected with sarcoptic mange showed an average weight reduction of 11.3% compared with their pre-release weights. Furthermore, 20% of the rescued animals exhibited severe hypothermia, which resulted in death the following day, suggesting that hypothermia is associated with a poor prognosis. The clinical symptoms observed in our study, such as crusting, hair loss, and dermatitis, are consistent with those reported in previous studies on sarcoptic mange in foxes [12]. As most foxes were found at an advanced stage of the disease, they exhibited clinical signs characteristic of chronic infection, such as severe crusting, rather than localized erythema. The hind legs, particularly the hock region, were the most commonly affected initial body parts, emphasizing the importance of inspecting this area when sarcoptic mange is suspected. Additionally, some animals who initially did not exhibit noticeable hair loss developed more pronounced symptoms as treatment progressed, with the shedding of keratinized epidermal layers becoming more apparent.

Previous studies of red foxes affected by sarcoptic mange have demonstrated that histopathological skin findings can vary depending on the types and stage of disease [4,13]. However, in the present study, these features were not observed, irrespective of disease severity. In addition, due to postmortem changes, only a limited number of skin tissue samples (*n* = 5) were suitable for histopathological examination, compared to the total number of carcasses examined (*n* = 12). Furthermore, a key limitation was the restriction of histopathological analysis to tissues with macroscopic lesions, preventing complete evaluation of all organ systems potentially affected by sarcoptic mange, as in previous Iberian ibex studies [40].

The CBC test results from our study, which showed distinct leukocytosis and anemia, are consistent with blood test results from other animals infected with sarcoptic mange, such as dogs, raccoon dogs, and southern hairy-nosed wombats [24,41,42,43]. However, in mange-infected fox species, neither wild-caught kit foxes nor experimentally infected red foxes showed significant erythrocyte reduction, which contrasts with our findings [13,26]. Moreover, this reduction in red blood cells was more significant in the group with diffuse skin lesions compared to those with localized lesions. Leukocytosis and anemia suggest a systemic response secondary to a chronic skin infection. In our study, the serum chemistry results showed significant hypoalbuminemia and increased globulin levels, with an insignificant increase in liver enzyme levels. Increased globulin levels are indicative of an enhanced immune response, a finding that was also observed in studies of kit foxes infected with sarcoptic mange [26]. Due to the lack of fractionated globulin analysis in the present study, it remains unclear which specific immunoglobulin components contributed to the observed increase. Postmortem examinations did not reveal pathological abnormalities in the liver, and most foxes had gastrointestinal parasites. This suggests that hypoalbuminemia is more indicative of protein loss through the gastrointestinal tract, chronic wasting disease, and nutritional deficiencies rather than liver dysfunction. Furthermore, in mange-infected foxes, BUN levels significantly increased, whereas creatinine levels decreased. The pattern of decreased creatinine levels observed in our study has also been noted in other species affected by sarcoptic mange, such as bobcats, southern hairy-nosed wombats, and common wombats [25,43,44]. In kit foxes, no differences were observed in serum biochemical test results based on the severity of skin lesions [26]. However, in our study, red foxes with severe skin lesions showed a significant decrease in creatinine levels compared to the localized infection group. In our study, postmortem examinations did not reveal pathological abnormalities in the kidneys, and phosphate levels were normal, suggesting that the increase in BUN levels was likely due to pre-renal azotemia rather than renal dysfunction. Decreased creatinine levels are likely related to muscle loss and nutritional deficiencies, which is consistent with the observed weight loss and hypoalbuminemia in the affected foxes. Furthermore, except for one fox that exhibited extreme hypoglycemia (27 mg/dL), all other foxes had blood glucose levels within normal ranges. This contrasts with the generally reported hypoglycemia in animals with sarcoptic mange [24,25,26,43,44].

Previous successful treatments for sarcoptic mange in wildlife typically involved administering ivermectin at doses of 200–400 µg/kg every 14 days [19]. However, a single dose of 300 µg/kg administered to red foxes has been reported as inadequate for effective treatment [23]. In our study, ivermectin was administered at a dose of 300 µg/kg weekly, with a minimum of two doses for localized infections and four doses for diffuse infections. Additionally, our treatment protocol included antibiotics and fluid therapy in all cases, as one study on raccoon dogs indicated that combining ivermectin with antibiotics and fluid therapy improved survival rates compared to ivermectin alone [45]. However, as most cases are accompanied by hypoalbuminemia and anemia, care must be taken to avoid overhydration. Although ivermectin has been associated with side effects, such as neurotoxicity and hepatotoxicity, the three foxes that died after receiving ivermectin in this study were already in a severe state, including profound hypothermia, which complicates the direct attribution of their deaths to ivermectin toxicity [46]. Ivermectin is a classic treatment with proven efficacy; however, it requires animal capture for treatment and may cause side effects in some cases. In contrast, the recently introduced oral medication fluralaner, used for pets, has a wider safety margin and a longer duration of action, lasting up to 12 weeks [47,48]. Fluralaner has demonstrated effectiveness in treating sarcoptic mange in wildlife, including the American black bear, raccoon dog, and wombat, suggesting that it may overcome some of the limitations of ivermectin, particularly its invasive properties [20,21,22]. In this study, we replaced ivermectin with fluralaner for ectoparasite prevention prior to release in 2019, anticipating a longer duration of efficacy with fluralaner compared to ivermectin (2 weeks for ivermectin vs. 12 weeks for fluralaner). However, since mange infections were detected at an average of 345.4 days post-release, more long-lasting preventive strategies need to be developed. Fluralaner was used solely for therapeutic purposes, not for prevention, in one fox with a localized infection, and clinical treatment effects were observed. Since only one mange-infected fox was treated with fluralaner, its effectiveness should be evaluated in additional cases, including those with diffuse lesions.

To successfully reintroduce the previously extinct red fox species into the wild in South Korea, it is essential not only for red foxes to adapt to their environment but also cope with diseases. Animals that have continuously lived in a region may have developed a certain degree of symbiosis with parasites, and a robust population density can help prevent diseases from progressing to extinction levels [17]. This study presents the first report on clinical cases of sarcoptic mange infection in reintroduced red foxes (*Vulpes vulpes*) in South Korea, offering valuable insights into the disease’s impact on conservation efforts and management strategies. Furthermore, our findings highlight that sarcoptic mange poses a significant challenge to red fox reintroduction and conservation efforts, affecting individual survival rates and population stability. Therefore, through this study, we aim to understand the disease dynamics related to sarcoptic mange in reintroduced red fox populations and provide empirical evidence for disease treatment and management strategies, ultimately improving the survival rates of reintroduced red foxes in South Korea and contributing to the establishment of stable populations.

## 5. Conclusions

As previous studies have shown, red foxes are highly susceptible to sarcoptic mange, and the foxes reintroduced to South Korea are also facing sarcoptic mange as the most significant threat among single diseases. The clinical cases of sarcoptic mange infections and related data, described for the first time in this study for the reintroduced foxes in South Korea, will serve as an important foundation for the effective treatment and prevention of sarcoptic mange in the future.

## Figures and Tables

**Table 1:** Sex, age, body weight, body temperature, skin lesions, antiparasitic treatment, treatment period, and results for 15 subsequent red fox (*Vulpes vulpes*) rescues with sarcoptic mange.

Case	Sex	Age (Years)	BW (kg)	BT (℃)	Skin Lesion	Antiparasitic	Treatment Period (Days)	Treatment Result
1	M	3	6.1	38.7	Local (hock)	Ivermectin	17	Complete recovery
2	M	2	5.3	37.2	Local (ear, elbow, hock)	Ivermectin	23	Complete recovery
3	F	4	6.3	37.9	Local (hind limb)	Ivermectin	13	Complete recovery
4	M	5	6.5	40.8	Local (hock)	Ivermectin	16	Complete recovery
5	F	<1	4.4	40.0	Local (ear, tail, hock)	Ivermectin	15	Complete recovery
6	F	4	5.2	37.4	Local (tail)	Ivermectin	15	Complete recovery
7	F	1	4.7	39.0	Local (muzzle, tail)	Fluralaner	16	Complete recovery
8	F	3	6.6	40.0	Diffuse	Ivermectin	28	Complete recovery
9	F	4	4.0	37.7	Diffuse	Ivermectin	26	Complete recovery
10	F	4	4.9	38.3	Diffuse	Ivermectin	25	Complete recovery
11	M	3	5.4	N/D ^a^	Diffuse	Ivermectin	1	Died
12	F	4	3.7	35.0	Diffuse	Ivermectin	1	Died
13	F	2	3.5	32.0	Diffuse	Ivermectin	1	Died
14	F	<1	4.8	40.0	Diffuse	Ivermectin	36	Complete recovery
15	M	2	6.0	40.2	Diffuse	Ivermectin	25	Complete recovery

F, female; M, male; BW, body weight; BT, body temperature. Body temperature was measured using a digital thermometer inserted into the rectum. ^a^ Not detected, below the range detectable with a digital thermometer. Case 3 and Case 8 were the same fox. The completely recovered foxes were either released or kept in enclosures within the facility.

**Table 2:** Sex, age, body weight, skin lesions, and pathological findings of red foxes (*Vulpes vulpes*) with sarcoptic mange that were found dead.

Case	Sex	Age (Years)	BW (kg)	Skin Lesion	Pathological Findings
1	F	<1	5.1	Local (hind limb)	(N) Internal organ rupture due to car crash, moderate crust formation in hind limb (HP) Severe chronic-active dermatitis with epidermal hyperplasia, hyperkeratosis, with intra-lesional mites
2	F	1	5.8	Local (hind limb)	(N) Severe thorax injury due to bite wound, mild alopecia and scaling in hind limb, moderate autolysis of abdominal organs (HP) N/A ^b^
3	F	3	4.6	Diffuse	(N) Severe crust formation, hemorrhagic enteritis (HP) Epidermal hyperplasia, hyperkeratosis, inflammatory cell infiltration in the dermis layer, no significant findings in small intestine
4	M	<1	2.98	Diffuse	(N) Severe crust formation, lung congestion, hemorrhagic enteritis with internal parasites (HP) Severe chronic-active dermatitis with epidermal hyperplasia, hyperkeratosis, with intra-lesional mites, mild-to-moderate interstitial pneumonia, moderate eosinophilic and lymphocytic enteritis with intra-lesional parasites
5	M	2	4.8	Diffuse	(N) Severe crust formation, hemorrhagic enteritis with internal parasites, multiple necrotic foci in the liver (HP) Moderate chronic eosinophilic superficial dermatitis with epidermal hyperplasia, hyperkeratosis with intra-lesional mites, no significant findings other than the general autolysis of the liver
6	M	6	4.3	Diffuse	(N) Severe crust formation, pulmonary congestion, hemorrhagic enteritis with internal parasites, petechial hemorrhages on the surface of the liver, kidney enlargement (HP) Orthokeratotic hyperkeratosis with intra-lesional mites, multifocal autolysis of the hepatic parenchyma, no significant findings other than the presence of basophilic material and cellular debris in some of the bronchioles, mild multifocal mineral deposition in the renal medulla
7	F	1	3.9	Diffuse	(N) Severe autolysis, internal parasites (HP) N/A ^b^
8	M	2	4.6	Diffuse	(N) Severe autolysis and severe crust formation (HP) N/A ^b^
9	M	1	N/D ^a^	Unknown	(N) Severe autolysis and severe crust formation (HP) N/A ^b^
10	F	1	N/D ^a^	Unknown	(N) Severe autolysis and severe crust formation (HP) N/A ^b^
11	F	4	N/D ^a^	Unknown	(N) Severe autolysis and severe crust formation (HP) N/A ^b^
12	F	1	N/D ^a^	Unknown	(N) Severe autolysis and severe crust formation (HP) N/A ^b^

F, female; M, male; BW, body weight; N, necropsy findings; HP, histopathologic findings. ^a^ Not detected; body weight could not be determined due to severe autolysis. ^b^ Not assessed; histopathologic examinations were not assessed due to autolysis.

**Table 3:** Mean, standard deviation (SD), median, and ranges for hematology and serum chemistry parameters of mange-infected and healthy red foxes (*Vulpes vulpes*).

Parameter	Mange-Infected fox					Captive Healthy fox				
	*n*	Mean (SD)		Median (Range)		*n*	Mean (SD)		Median (Range)	
**White blood cells (10^9^/** **L** **) ^a,2^**	15	23.5	(14.6)	**18.5**	**(9.3** **–** **54.7)**	208	7.3	(2.5)	**6.8**	**(2.2** **–** **14.8)**
**Lymphocyte (10^9^/** **L** **) ^b,1^**	15	**7.0**	**(5.4)**	5.8	(1.1–21.5)	208	**2.1**	**(1.0)**	1.9	(0.3–5.3)
**Monocyte (10^9^/** **L** **) ^b,1^**	15	**1.9**	**(1.4)**	1.4	(0.5–4.9)	208	**0.5**	**(0.2)**	0.5	(0.2–1.1)
**Granulocyte (10^9^/** **L** **) ^a,2^**	15	14.7	(10.8)	**11.5**	**(3.1** **–** **40.3)**	208	4.7	(1.9)	**4.4**	**(1.1** **–** **10.7)**
Lymphocyte (%) ^2^	15	30.2	(18.0)	22.7	(8.5–65.0)	208	28.5	(10.3)	27.6	(6.5–58.6)
Monocyte (%) ^1^	15	8.1	(2.4)	8.7	(3.5–11.3)	208	7.3	(2.0)	6.9	(3.8–13.4)
Granulocyte (%) ^1^	15	61.7	(19.1)	68.5	(26.3–82.0)	208	64.2	(10.6)	64.7	(33.7–86.5)
**Eosinophil (%) ^a,1^**	15	**15.5**	**(8.2)**	13.2	(6.5–32.4)	208	**8.9**	**(5.7)**	7.6	(1.0–29.3)
**Red blood cells (10^12^/** **L** **) ^a,1^**	15	**6.1**	**(2.4)**	5.8	(2.1–10.3)	208	**9.9**	**(0.9)**	9.9	(7.1–12.1)
**Hemoglobin (g/** **L** **) ^a,1^**	15	**112.3**	**(41.6)**	107.0	(30–181)	208	**174.6**	**(15.8)**	174.0	(128–215)
**Hematocrit (L/L) ^a,1^**	15	**0.30**	**(0.09)**	0.29	(0.13–0.45)	208	**0.46**	**(0.39)**	0.46	(0.34–0.55)
**Platelet (10^9^/** **L** **) ^a,2^**	13	1089.3	(712.9)	**810.0**	**(527** **–** **3035)**	208	536.2	(241.6)	**463.0**	**(196** **–** **1606)**
Total protein (g/L) ^2^	15	69.0	(15.8)	67.0	(50–120)	203	65.2	(6.4)	65.0	(50–83)
**Albumin (g/** **L** **) ^a,2^**	14	26.1	(10.4)	**24.5**	**(16** **–** **60)**	203	30.0	(2.7)	**30.0**	**(24** **–** **37)**
**Globulin (g/** **L** **) ^a,1^**	13	**41.2**	**(5.4)**	43.0	(31–50)	203	**35.3**	**(5.2)**	35.0	(25–48)
Glucose (mmol/L) ^1^	15	7.5	(2.7)	7.4	(1.5–14.2)	203	7.7	(1.3)	7.7	(4.8–11.3)
Alanine aminotransferase (U/L) ^2^	15	236.5	(247.2)	117.0	(63–1000)	203	123.2	(50.2)	113.0	(46–297)
Alkaline phosphatase (U/L) ^2^	15	159.4	(443.2)	40.0	(13–1759)	200	37.6	(13.2)	36.0	(11–85)
**Blood urea nitrogen (mmol/** **L** **) ^c,2^**	15	12.2	(9.4)	**9.6**	**(4.6** **–40.3** **)**	203	7.6	(2.4)	**7.1**	**(3.6** **–17.1** **)**
**Creatinine (** **μ** **mol/** **L** **) ^a,1^**	14	**44.8**	**(15.3)**	44.2	(17.7–61.9)	203	**67.8**	**(14.4)**	70.7	(35.4–114.9)
Calcium (mmol/L) ^1^	10	2.0	(0.2)	2.1	(1.7–2.2)	199	2.3	(0.1)	2.3	(2.1–2.6)
Phosphate (mmol/L) ^1^	10	1.4	(0.4)	1.5	(0.7–1.9)	199	1.4	(0.3)	1.4	(0.9–2.2)
Sodium (mmol/L) ^1^	14	152.6	(8.3)	152.5	(136–168)	153	152.1	(4.2)	152.0	(142–163)
Potassium (mmol/L) ^2^	14	4.3	(1.7)	4.0	(3.2–10.0)	154	3.8	(0.3)	3.8	(2.9–4.4)
Chloride (mmol/L) ^1^	14	114.9	(5.9)	113.0	(107–128)	153	117.6	(2.9)	118.0	(110–126)

^a^ *p* < 0.001; ^b^ *p* < 0.01; ^c^ *p* < 0.05. ^1^ Indicates the parameters of the mange-infected foxes that follow a normal distribution; Student’s *t*-test analysis was used to analyze differences. ^2^ Indicates the parameters of the mange-infected foxes that do not follow a normal distribution; Mann–Whitney U test analysis was used to analyze differences. The parameters showing statistically significant differences and their corresponding statistical values are highlighted in bold.

## Data Availability

The data presented in this study are available upon request from the corresponding author, after approval of the National Park Institute for Wildlife Conservation.

## Abbreviations

The following abbreviations are used in this manuscript:

CBC	Complete blood count
BUN	Blood urea nitrogen

## References

[1] Escobar L.E., Carver S., Cross P.C., Rossi L., Almberg E.S., Yabsley M.J., Niedringhaus K.D., Van Wick P., Dominguez-Villegas E., Gakuya F. (2022). Sarcoptic Mange: An Emerging Panzootic in Wildlife. Transbound. Emerg. Dis..

[2] Little S.E., Davidson W.R., Howerth E.W., Rakich P.M., Nettles V.F. (1998). Diseases Diagnosed in Red Foxes from the Southeastern United States. J. Wildl. Dis..

[3] Soulsbury C.D., Iossa G., Baker P.J., Cole N.C., Funk S.M., Harris S. (2007). The Impact of Sarcoptic Mange *Sarcoptes scabiei* on the British Fox *Vulpes vulpes* Population. Mammal Rev..

[4] Nimmervoll H., Hoby S., Robert N., Lommano E., Welle M., Ryser-Degiorgis M.P. (2013). Pathology of Sarcoptic Mange in Red Foxes (*Vulpes vulpes*): Macroscopic and Histologic Characterization of Three Disease Stages. J. Wildl. Dis..

[5] Kołodziej-Sobocińska M., Zalewski A., Kowalczyk R. (2014). Sarcoptic Mange Vulnerability in Carnivores of the Białowieża Primeval Forest, Poland: Underlying Determinant Factors. Ecol. Res..

[6] Perrucci S., Verin R., Mancianti F., Poli A. (2016). Sarcoptic Mange and Other Ectoparasitic Infections in a Red Fox (*Vulpes vulpes*) Population from Central Italy. Parasite Epidemiol. Control.

[7] Niedringhaus K.D., Brown J.D., Sweeley K.M., Yabsley M.J. (2019). A Review of Sarcoptic Mange in North American Wildlife. Int. J. Parasitol. Parasites Wildl..

[8] Browne E., Driessen M.M., Cross P.C., Escobar L.E., Foley J., López-Olvera J.R., Niedringhaus K.D., Rossi L., Carver S. (2022). Sustaining Transmission in Different Host Species: The Emblematic Case of *Sarcoptes scabiei*. BioScience.

[9] Bornstein S., Mörner T., Samuel W.M. (2001). Sarcoptes scabiei and Sarcoptic Mange. Parasitic Diseases of Wild Mammals.

[10] Pence D.B., Ueckermann E. (2002). Sarcoptic Mange in Wildlife. Rev. Sci. Tech..

[11] Mörner T., Christensson D. (1984). Experimental Infection of Red Foxes (*Vulpes vulpes*) with *Sarcoptes scabiei* var. *vulpes*. Vet. Parasitol..

[12] Bornstein S., Zakrisson G., Thebo P. (1995). Clinical Picture and Antibody Response to Experimental *Sarcoptes scabiei* var. *vulpes* Infection in Red Foxes (*Vulpes vulpes*). Acta Vet. Scand..

[13] Little S.E., Davidson W.R., Rakich P.M., Nixon T.L., Bounous D.I., Nettles V.F. (1998). Responses of Red Foxes to First and Second Infection with *Sarcoptes scabiei*. J. Wildl. Dis..

[14] Baker P.J., Funk S.M., Harris S., White P.C. (2000). Flexible Spatial Organization of Urban Foxes, *Vulpes vulpes*, Before and During an Outbreak of Sarcoptic Mange. Anim. Behav..

[15] Lindström E.R., Andrén H., Angelstam P., Cederlund G., Hörnfeldt B., Jäderberg L., Lemnell P.A., Martinsson B., Sköld K., Swenson J.E. (1994). Disease Reveals the Predator: Sarcoptic Mange, Red Fox Predation, and Prey Populations. Ecology.

[16] Davidson R.K., Bornstein S., Handeland K. (2008). Long-Term Study of *Sarcoptes scabiei* Infection in Norwegian Red Foxes (*Vulpes vulpes*) Indicating Host/Parasite Adaptation. Vet. Parasitol..

[17] Willebrand T., Samelius G., Walton Z., Odden M., Englund J. (2022). Declining Survival Rates of Red Foxes *Vulpes vulpes* During the First Outbreak of Sarcoptic Mange in Sweden. Wildlife Biol..

[18] Henriksen P., Diets H.H., Henriksen S.A., Gjelstrup P. (1993). Sarcoptic Mange in Red Fox in Denmark. A Short Report. Dan. Vettidsskr..

[19] Rowe M.L., Whiteley P.L., Carver S. (2019). The Treatment of Sarcoptic Mange in Wildlife: A Systematic Review. Parasit. Vectors.

[20] Hyun J.E., Jang H.K., Hwang C.Y., Yeon S.C. (2019). Clinical Efficacy of Orally Administered Fluralaner for Treatment of Scabies in Six Free-Rearing Raccoon Dogs (*Nyctereutes procyonoides*). Vet. Dermatol..

[21] Van Wick M., Hashem B. (2019). Treatment of Sarcoptic Mange in an American Black Bear (*Ursus americanus*) with a Single Oral Dose of Fluralaner. J. Wildl. Dis..

[22] Wilkinson V., Takano K., Nichols D., Martin A., Holme R., Phalen D., Mounsey K., Charleston M., Kreiss A., Pye R. (2021). Fluralaner as a Novel Treatment for Sarcoptic Mange in the Bare-Nosed Wombat (*Vombatus ursinus*): Safety, Pharmacokinetics, Efficacy and Practicable Use. Parasit. Vectors.

[23] Newman T.J., Baker P.J., Harris S. (2002). Nutritional Condition and Survival of Red Foxes with Sarcoptic Mange. Can. J. Zool..

[24] Kido N., Kamegaya C., Omiya T., Wada Y., Takahashi M., Yamamoto Y. (2011). Hematology and Serum Biochemistry in Debilitated, Free-Ranging Raccoon Dogs (*Nyctereutes procyonoides*) Infested with Sarcoptic Mange. Parasitol. Int..

[25] Serieys L.E., Foley J., Owens S., Woods L., Boydston E.E., Lyren L.M., Poppenga R.H., Clifford D.L., Stephenson N., Rudd J. (2013). Serum Chemistry, Hematologic, and Post-Mortem Findings in Free-Ranging Bobcats (*Lynx rufus*) with Notoedric Mange. J. Parasitol..

[26] Rudd J., Clifford D., Richardson D., Cypher B., Westall T., Kelly E., Foley J. (2019). Hematologic and Serum Chemistry Values of Endangered San Joaquin Kit Foxes (*Vulpes macrotis mutica*) with Sarcoptic Mange. J. Wildl. Dis..

[27] Won C., Smith K.G. (1999). History and Current Status of Mammals of the Korean Peninsula. Mammal Rev..

[28] Yu J.N., Han S.H., Kim B.H., Kryukov A.P., Kim S., Lee B.Y., Kwak M. (2012). Insights into Korean Red Fox (*Vulpes vulpes*) Based on Mitochondrial Cytochrome b Sequence Variation in East Asia. Zool. Sci..

[29] Jo Y.S., Baccus J.T., Koprowski J.L. (2018). Red Fox. Mammals of Korea.

[30] Lee W.K., Cho B.K. (1995). Taxonomical Approach to Scabies Mites of Human and Animals and Their Prevalence in Korea. Korean J. Parasitol..

[31] Eo K.Y., Kwon O.D., Shin N.S., Shin T., Kwak D. (2008). Sarcoptic Mange in Wild Raccoon Dogs (*Nyctereutes procyonoides*) in Korea. J. Zoo Wildl. Med..

[32] Park D.S., Choi J., Kim H.J., Kim J.Y., Kim M.H., Lee J.Y., Moon J.C., Park H.B., Park K.M., Yun J.H. (2022). Two Cases of Mange Mite (*Sarcoptes scabiei*) Infestation in Long-Tailed Goral (*Naemorhedus caudatus*) in Republic of Korea. Korean J. Parasitol..

[33] Lucio-Forster A., Lejeune M. (2020). Diagnostic Parasitology. Georgis’ Parasitology for Veterinarians.

[34] Kelly T.R., Sleeman J.M. (2003). Morbidity and Mortality of Red Foxes (*Vulpes vulpes*) and Gray Foxes (*Urocyon cinereoargenteus*) Admitted to the Wildlife Center of Virginia, 1993–2001. J. Wildl. Dis..

[35] Cypher B.L., Rudd J.L., Westall T.L., Woods L.W., Stephenson N., Foley J.E., Richardson D., Clifford D.L. (2017). Sarcoptic Mange in Endangered Kit Foxes (*Vulpes macrotis mutica*): Case Histories, Diagnoses, and Implications for Conservation. J. Wildl. Dis..

[36] Storm G.L., Andrews R.D., Phillips R.L., Bishop R.A., Siniff D.B., Tester J.R. (1976). Morphology, Reproduction, Dispersal, and Mortality of Mild Western Red Fox Population. Wildl. Monogr..

[37] Schalk G., Forbes M.R. (1997). Male Biases in Parasitism of Mammals: Effects of Study Type, Host Age, and Parasite Taxon. Oikos.

[38] Devenish-Nelson E.S., Richards S.A., Harris S., Soulsbury C., Stephens P.A. (2014). Demonstrating Frequency-Dependent Transmission of Sarcoptic Mange in Red Foxes. Biol. Lett..

[39] Martin A.M., Fraser T.A., Lesku J.A., Simpson K., Roberts G.L., Garvey J., Polkinghorne A., Burridge C.P., Carver S. (2018). The Cascading Pathogenic Consequences of *Sarcoptes scabiei* Infection That Manifest in Host Disease. R. Soc. Open Sci..

[40] Espinosa J., Ráez-Bravo A., López-Olvera J.R., Pérez J.M., Lavín S., Tvarijonaviciute A., Cano-Manuel F.J., Fandos P., Soriguer R.C., Granados J.E. (2017). Histopathology, microbiology and the inflammatory process associated with *Sarcoptes scabiei* infection in the Iberian ibex, *Capra pyrenaica*. Parasites Vectors.

[41] Arlian L.G., Morgan M.S., Rapp C.M., Vyszenski-Moher D.L. (1995). Some Effects of Sarcoptic Mange on Dogs. J. Parasitol..

[42] Beigh S.A., Soodan J.S., Singh R., Raina R. (2013). Plasma Zinc, Iron, Vitamin A and Hematological Parameters in Dogs with Sarcoptic Mange. Isr. J. Vet. Med..

[43] Ruykys L., Breed B., Schultz D., Taggart D. (2013). Effects and Treatment of Sarcoptic Mange in Southern Hairy-Nosed Wombats (*Lasiorhinus latifrons*). J. Wildl. Dis..

[44] Skerratt L.F., Middleton D., Beveridge I. (1999). Distribution of Life Cycle stages of *Sarcoptes scabiei* var *wombati* and Effects of Severe Mange on Common Wombats in Victoria. J. Wildl. Dis..

[45] Kido N., Omiya T., Kamegaya C., Wada Y., Takahashi M., Yamamoto Y. (2014). Effective Treatment for Improving the Survival Rate of Raccoon Dogs Infected with *Sarcoptes scabiei*. J. Vet. Med. Sci..

[46] Salman M., Abbas R.Z., Mehmood K., Hussain R., Shah S., Faheem M., Zaheer T., Abbas A., Morales B., Aneva I. (2022). Assessment of Avermectins-Induced Toxicity in Animals. Pharmaceuticals.

[47] Rohdich N., Roepke R.K., Zschiesche E. (2014). A Randomized, Blinded, Controlled and Multi-Centered Field Study Comparing the Efficacy and Safety of Bravecto™(Fluralaner) Against Frontline™(Fipronil) in Flea-and Tick-Infested Dogs. Parasites Vectors.

[48] Walther F.M., Allan M.J., Roepke R.K., Nuernberger M.C. (2014). Safety of Fluralaner Chewable Tablets (Bravecto TM), a Novel Systemic Antiparasitic Drug, in Dogs after Oral Administration. Parasites Vectors.

